# Antigen receptor signalling: a distinctive role for the p110δ isoform of PI3K

**DOI:** 10.1016/j.it.2006.12.007

**Published:** 2007-02

**Authors:** Klaus Okkenhaug, Khaled Ali, Bart Vanhaesebroeck

**Affiliations:** 1 Laboratory of Lymphocyte Signalling and Development, Babraham Institute, Cambridge, UK, CB2 4AT; 2 Ludwig Institute for Cancer Research, London, UK, W1W 7BS; 3 Department of Biochemistry and Molecular Biology, University College London, London, UK, WC1E 6BT

## Abstract

The activation of antigen receptors triggers two important signalling pathways originating from phosphatidylinositol(4,5)-bisphosphate [PtdIns(4,5)*P*_2_]. The first is phospholipase Cγ (PLCγ)-mediated hydrolysis of PtdIns(4,5)*P*_2_, resulting in the activation of Ras, protein kinase C and Ca^2+^ flux. This culminates in profound alterations in gene expression and effector-cell responses, including secretory granule exocytosis and cytokine production. By contrast, phosphoinositide 3-kinases (PI3Ks) phosphorylate PtdIns(4,5)*P*_2_ to yield phosphatidylinositol(3,4,5)-trisphosphate, activating signalling pathways that overlap with PLCγ or are PI3K-specific. Pathways that are PI3K-specific include Akt-mediated inactivation of Foxo transcription factors and transcription-independent regulation of glucose uptake and metabolism. The p110δ isoform of PI3K is the main source of PI3K activity following antigen recognition by B cells, T cells and mast cells. Here, we review the roles of p110δ in regulating antigen-dependent responses in these cell types.

## Introduction

Leukocytes express a repertoire of receptors to recognize and bind to extracellular antigen. This binding can be direct, as in the case of the B-cell antigen receptor (BCR) and the T-cell antigen receptor (TCR), or indirect, namely through receptors that interact with the Fc portion of immunoglobulins (Ig). The latter include the high affinity receptor for IgE (termed FcɛRI) on mast cells and basophils, and the FcγR for IgG on phagocytes, NK cells and B cells. PI3K activity has been implicated in the signalling of all types of antigen receptors.

## PI3K isoforms

Mammals have eight isoforms of phosphoinositide 3-kinase (PI3K), divided into three classes [1]. Class IA PI3Ks signal downstream of tyrosine kinases and Ras [1]. Class IA p110 catalytic subunits (p110α, p110β and p110δ) are constitutively bound to an SH2 domain-containing adaptor protein, of which there are five species in mammals (p85α, p55α, p50α, p85β and p55γ; often referred to as ‘p85s’). None of the distinct p85s has been shown to be enriched in leukocytes and, *in vitro*, each p85 can interact with each p110 species. The Src homology 2 (SH2) domains of p85s are thought to bind preferentially to tyrosine (Tyr)-based motifs known as Y_p_xxM (Yp, phosphoTyr; M, methionine; x, any amino acid).

The p110γ PI3K, which belongs to the class IB subset of PI3Ks, also phosphorylates phosphatidylinositol(4,5)-bisphosphate [PtdIns(4,5)*P*_2_] but is activated by G protein-coupled receptors (GPCRs) instead of through tyrosine kinases. p110γ is also regulated by Ras [2]. In immune cells, p110γ is activated mainly by chemokines and by adenosine [3–5]. Therefore, p110γ might generate a distinct pool of phosphatidylinositol (3,4,5)-trisphosphate [PtdIns(3,4,5)*P*_3_] in activated cells.

The class II and class III PI3Ks have not been implicated in immune signalling, and are not considered further here.

## PI3K isoforms and antigen receptor signalling

Of the three class IA PI3K catalytic isoforms (p110α, p110β and p110δ), p110δ seems to have evolved to regulate PI3K-dependent processes in immune cells, most probably in part related to its high expression in these cells compared with most other cell types. Lymphocyte and mast-cell antigen receptor-dependent PI3K signalling is compromised in mice in which p110δ has been inactivated by gene deletion [6,7], point mutation (p110δ^D910A^) [8] or small-molecule p110δ inhibitors [9–11]. p110δ seems to be less crucial in IgG-based antigen receptor (FcγR)-mediated phagocytosis in macrophages, where the p110β isoform seems to be more important [12]. It should be noted that p110α and p110β are also expressed in leukocytes, and together can contribute up to 50% of the total p85-associated PI3K activity in some leukocytes [8–10,14]. Therefore, although we emphasize the predominant role of p110δ in antigen receptor signalling in this review, it is possible that roles for p110α and p110β in this signalling context will be uncovered with the ongoing development of conditional knockout mice and selective inhibitors for these PI3K isoforms.

## Conversion of PtdIns(4,5)*P*_2_ downstream of antigen receptors

**Figure 1:**
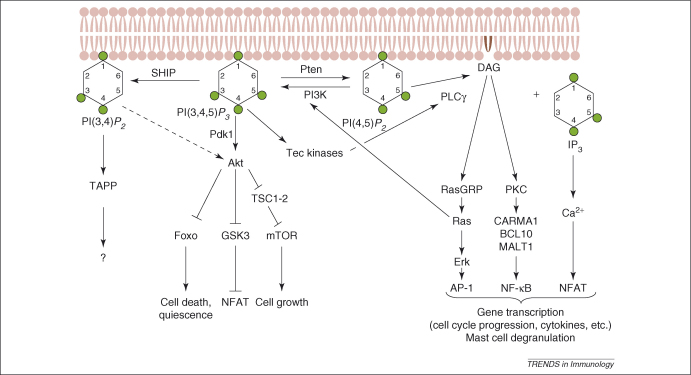
Metabolism of PtdIns(4,5)*P*_2_ by PLCγ and PI3K. PLCγ hydrolyses PtdIns(4,5)*P*_2_ to yield Ins(1,4,5)*P*_3_ and DAG, both of which function as signalling molecules. Ins(1,4,5)P_3_ stimulates the release of Ca^2+^ from the ER into the cytosol, which triggers the nuclear translocation of NFAT. DAG binds to and activates RasGRP, which stimulates Ras and the Erk pathway, leading to AP-1-dependent transcription. Ras also binds to p110 and contributes to optimal PI3K activation. DAG binds to and activates PKC, which activates NF-κB through CARMA1, BCL10 and MALT1. By contrast, PI3K phosphorylates PtdIns(4,5)*P*_2_ at position 3 to produce the membrane phosphoinositol lipid PtdIns(3,4,5)*P*_3_. PtdIns(3,4,5)*P*_3_ functions as an anchor and cofactor for proteins with PtdIns(3,4,5)*P*_3_-binding PH domains such as Akt, Tec family kinases, and various GEFs and GAPs. Pdk1 is required to co-activate Akt. Akt phosphorylates and inactivates Foxo and GSK3. GSK3 can phosphorylate and inactivate NFAT. Akt stimulates mTOR through Tsc1 and Tsc2. Tec kinases can phosphorylate PLCγ and contribute to its optimal activity. PI3K signalling is antagonised by the Pten phosphoinositide phosphatase, which removes the 3-phosphate, and the SHIP phosphatase, which removes the 5-phosphate. The role of PI(3,4)P_2_-binding proteins is still unknown. Although PLCγ and PI3K generate mutually exclusive second messenger signalling molecules, several of the pathways activated by these second messengers interact, and the signals are further integrated by the cell to promote gene transcription, cell growth and differentiation. p110δ seems to be the principal PI3K isoform in the context of antigen receptor signaling; however, p110α and p110β are also expressed in lymphocytes but their roles in antigen receptor signalling are unknown. Abbreviations: BCL10, B cell lymphoma 10; CARMA1, caspase recruitment domain (CARD)-containing membrane-associated guanylate kinase (MAGUK) protein 1; IP_3_, inositol(1,4,5)-trisphosphate; MALT1, mucosa-associated lymphoid tissue lymphoma translocation protein 1; PI, phosphatidylinositol.

Antigen receptor stimulation initiates the activation of Src and Syk family Tyr kinases, resulting in Tyr phosphorylation of adaptor proteins and the activation of two PtdIns(4,5)*P*_2_-based signalling pathways (Figure 1).

The first of these is mediated by phospholipase C (PLC)γ, which hydrolyses PtdIns(4,5)*P*_2_ to generate diacylglycerol (DAG) and inositol(1,4,5)-trisphosphate [Ins(1,4,5)*P*_3_] (Figure 1). The water-soluble Ins(1,4,5)*P*_3_ triggers a biphasic Ca^2+^ response, initially by inducing Ca^2+^ release from the endoplasmic reticulum, followed by Ca^2+^-dependent opening of plasma membrane channels to enable sustained Ca^2+^ influx and the nuclear translocation of NFAT (nuclear factor of activated T cells) transcription factors [15]. DAG activates Ras guanyl nucleotide-releasing proteins (RasGRPs, which bind to Ca^2+^ and DAG) and isoforms of protein kinase C (PKC), which initiate the Ras–Erk–AP-1 and nuclear factor (NF)-κB signalling pathways, respectively [16–20]. Hence, the hydrolysis of PtdIns(4,5)*P*_2_ by PLCγ is sufficient to initiate the activation of three transcription factor families (Figure 1) that coordinately regulate the expression of a vast number of genes involved in cytokine production, cell division and differentiation [21,22]. Antigen receptor cross-linking also activates PLCγ-dependent pathways involved in promoting the release of preformed mast-cell secretory granules, which promote the early symptoms of allergic hypersensitivity reactions [19].

Class I PI3Ks mediate an alternative conversion of PtdIns(4,5)*P*_2_, namely by phosphorylating this lipid to PtdIns(3,4,5)*P*_3_ (Figure 1). In contrast to its precursor, PtdIns(3,4,5)*P*_3_ is resistant to PLC-mediated hydrolysis and, instead, signals at the plasma membrane by functioning as docking sites for pleckstrin homology (PH) domains that are present in several proteins [1]. These PtdIns(3,4,5)*P*_3_ targets include protein kinases (such as Pdk1, Akt and Tec kinases), adaptor proteins (such as Gab2), and GTPase activating proteins (GAPs) and guanine nucleotide exchange factors (GEFs) for small GTPases (such as P-Rex, ARAP, SWAT-70, IBP and Vav) [1,23,24]. PtdIns(3,4,5)*P*_3_ binding induces the rapid recruitment of these proteins to the membrane in response to PI3K activation and/or alteration of their conformation or activity [25,26]. This abundance of downstream targets link PI3K to its well-established roles in cell cycle progression, growth, prevention of apoptosis, cell migration, differentiation and secretory granule exocytosis.

PtdIns(3,4,5)*P*_3_ is a substrate for lipid phosphatases, amongst which the 3-phosphatase Pten and the 5-phosphatase SHIP are most widely studied and have important roles in antagonising PI3K signalling [27,28]. SHIP converts PtdIns(3,4,5)*P*_3_ to PtdIns(3,4)*P*_*2*_, which can bind to a limited set of PH domains, including those of tandem PH domain-containing protein (TAPP)1 and TAPP2 [29] (Figure 1)_._

## PI3K coupling to the antigen receptor

**Figure 2:**
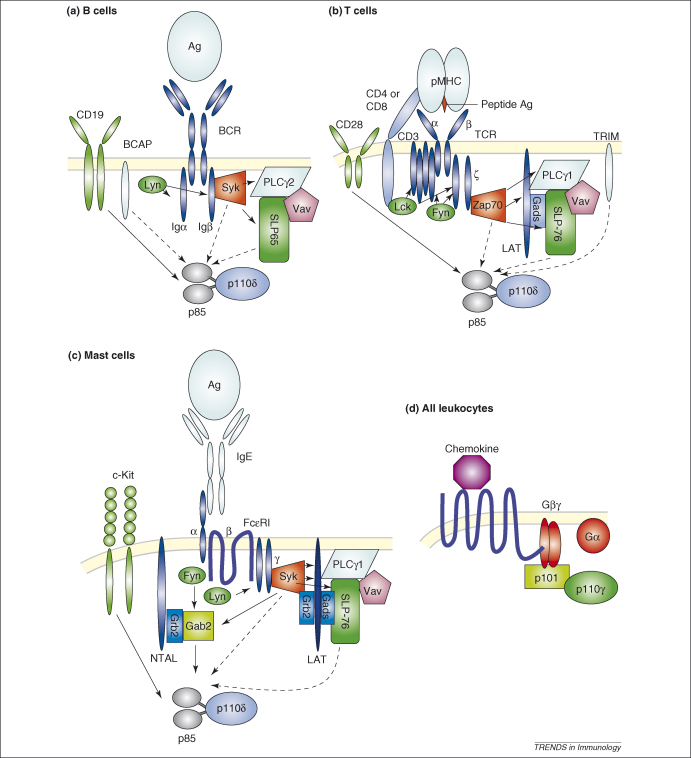
Antigen receptor complexes and p110δ antigen receptor signalling in different cell types has key commonalities, the most crucial of which is the phosphorylation of ITAM motifs found in proteins that are noncovalently associated with the polypeptides that bind to the antigen or antibody–antigen complexes. Src-family kinases phosphorylate these ITAM motifs, thus providing docking sites for Syk kinases. Syk kinases phosphorylate PLCγ, resulting in its activation, in addition to the recruitment of various cellular and membrane-bound adaptor proteins, such as LAT, Gab2, SLP-76 (in T cells and mast cells) and SLP-65 (in B cells) that nucleate larger signalling complexes [18,19]. The upstream activators of PI3K in the context of antigen receptor signalling have not been definitively defined, and possible links with the SH2 domains of p85 are indicated by dashed arrows. Phosphorylation-independent interactions of PI3K with upstream signalling molecules are not shown. Note that the antigens are shown as monomers for illustrative purposes. In reality, dimers or oligomers of the ligands and receptors are required to trigger the signalling cascades shown. **(a)** BCR signalling. Lyn-dependent phosphorylation of Igα and Igβ ITAM motifs results in the recruitment of Syk and the phosphorylation of SLP-65. Several proteins in the BCR receptor complex have been implicated in binding to p85. BCAP is a transmembrane adaptor protein with YxxM motifs which becomes phosphorylated by Syk upon BCR activation. BCAP has been shown to regulate PI3K signalling in DT40 chicken cells but was found to be not required for PI3K signalling in primary mouse B cells [40,87]. Similarly, Vav had been proposed to lie upstream of PI3K signalling [56]; however, BCR crosslinking of primary Vav-deficient B cells results in normal Akt phosphorylation (although Akt phosphorylation in response to BCR and CD19 coligation was Vav-dependent) [51]. Other candidates that link PI3K to the activated BCR include Gab1, non T-cell activation linker (NTAL) and LAT. However, many of these interactions have only been identified in cell lines, and in several cases, their roles in PI3K signalling downstream of the BCR have not been confirmed in mice [88,89]. **(b)** TCR signalling. Lck and Fyn phosphorylate ITAM motifs resulting in the recruitment of ZAP-70, which phosphorylates LAT. TRIM is a transmembrane adaptor protein that associates with the TCR and becomes phosphorylated on YxxM motifs. However, TRIM knockout mice show enhanced instead of impaired Akt phosphorylation [39]. Vav has an important role in regulating Akt and Foxo phosphorylation. Although the exact biochemical link between Vav and PI3K is not clearly defined, it may reflect a more general role for Vav in assembling LAT complexes [71,90,91]. CD28 can bind to the SH2 domains of p85 directly; however, CD28 can provide potent costimulatory signals independently of its association with PI3K [30]. **(c)** FcɛRI signalling. Lyn phosphorylates ITAM motifs in the β and γ chains of the FcɛRI resulting in the recruitment of Syk, which phosphorylates GAB2 and LAT, inducing two parallel pathways through GAB2–PI3K and LAT–PLCγ. The link shown between NTAL and GAB2 is hypothetical. c-Kit can bind to p85 directly and can potentiate FcɛRI-stimulated degranulation. **(d)** Chemokine receptor signalling. The p110γ heterodimer binds to the Gβγ subunit released from Gα following GPCR stimulation with agonists such as chemokines. Despite the potent activation of p110γ by chemokines, p110γ seems to have a minor role in promoting lymphocyte chemotaxis [3]. Instead, p110γ might promote the survival of developing thymocytes and memory T cells [92].

The details of how PI3Ks are linked to antigen receptor-associated signalling complexes are still vague [30–32]. What is clear is that PtdIns(3,4,5)*P*_3_ accumulation occurs extremely rapidly following antigen recognition, which suggests that the association between the antigen receptor signalling complexes and PI3K is tightly coupled to the initial tyrosine kinase signals [29,33–38]. Several of the adaptor molecules in the antigen receptor complex contain canonical YxxM motifs, although the role of some of these, including B-cell adaptor protein (BCAP) and T-cell receptor interacting molecule (TRIM), is still unclear [39,40] (Figure 2). An intriguing recent study suggested that p85 can bind to Syk and zeta-chain associated protein kinase of 70 kDa (ZAP-70) directly through non-canonical Tyr-based motifs [41]. There is also evidence that p85 can bind to SH2 domain-containing leukocyte phosphoprotein of 76 kD (SLP76), again through noncanonical motifs [42]. The SH3 domains and proline-rich regions present in p85s also offer scope for phosphorylation-independent binding to signalling proteins. If these interactions can be confirmed under physiologically relevant conditions, then we might need to cast the net wider in considering potential players in the recruitment of p85–p110 heterodimers to antigen receptor signalling complexes.

Given that p85 species seem to have no binding preference for specific p110 PI3K isoforms, it is anticipated that this type of recruitment would not favour p110δ over p110α and p110β for association with the antigen receptor. However, in addition to binding to p85, the p110 subunits can also bind to Ras-GTP [1]. Ras signalling is important in antigen receptor signalling [16]. Evidence is accumulating that each p110 isoform has a distinct binding capacity to Ras, or shows a binding preference for specific Ras isoforms (reviewed in Ref. [1]). In one study, p110α was found to become activated by most Ras isoforms, whereas p110δ became activated selectively by R-Ras and Tc21; p110β did not become activated by Ras at all [43]. Hence, the differential usage of Ras isoforms could contribute to selective recruitment and/or activation of class IA PI3K isoforms. Therefore, the potential role of Ras in regulating the activity of different PI3K isoforms in the context of antigen receptor signalling warrants further investigation.

## p110δ in B-cell development and function

p110δ deficiency† does not have a major impact on the early development of B cells in the bone marrow [6–8]. By contrast, the development of mature B cells in the spleen and in pleural cavities is strongly affected. Thus, the number of follicular B2 cells is reduced to <50% of normal numbers. In addition, the development of peritoneal B1 cells and marginal-zone B cells is almost completely blocked [30,31,44].

The *in vitro* proliferation of B cells triggered by antibody-mediated crosslinking (through anti-IgM) of the BCR crucially depends on p110δ activity [6–9]. Deletion or inactivation of p110δ or p85α largely ablates BCR-induced phosphorylation of Akt, Foxo and protein kinase D, and results in reduced Ca^2+^ flux, impaired cell cycle progression and reduced glucose metabolism [9,45–50]. Interleukin (IL)-4-dependent survival is also compromised in the absence of p110δ activity [9]. CD40 and lipopolysaccharide signalling is less dependent on PI3K signalling, so activated T cells and selected pathogens might still stimulate B-cell responses even in the context of strongly attenuated BCR signalling [8,51]. PI3K had initially been suggested to be part of a BCR-associated signalosome based on the similar phenotypes of Btk, p85α, p110δ, and PLCγ knockout mice. In this model, the principal role for PI3K would be to promote PLCγ activity [52]. Although the role of PI3K in regulating PLCγ is well established, recent evidence shows that PLCγ and PI3K pathways also function in parallel (Figure 1). Thus, p85α–Btk and p85α–PLC-γ2 double-knockout mice show much more dramatic phenotypes than any of the single knockout mice and provide evidence for PLCγ-independent functions for PI3K [53,54].

PI3K activation in B cells can be enhanced by the coordinated engagement of the BCR and its co-receptor CD19, which contains YxxM recruitment motifs for PI3K [55]. This enables added sensitivity of the B cells to antigens that are coated with complement. In this context, Vav is required for optimal PI3K responses, perhaps reflecting a role for Vav in coordinating crosstalk between these receptors by assembling larger signalling complexes [56,57]. The B-cell phenotypes observed in p85α-knockout and p110δ-deficient mice largely overlap with those observed in CD19-deficient mice. The key role for PI3K in the context of CD19 function is further evidenced by the failure to rescue a normal B-cell phenotype in CD19 knockout mice by the transgenic expression of a CD19 mutant that cannot bind to PI3K [58]. Moreover, Pten-deficiency can partially revert the effect of CD19 deficiency, presumably by lowering the signalling threshold required to initiate PI3K signalling [59]. Co-ligation of the BCR and FcγRIIB, a low affinity receptor for IgG, also forms the basis for modulating PtdIns(3,4,5)*P*_3_ signals downstream of the BCR through the recruitment of SHIP, which converts PtdIns(3,4,5)*P*_3_ into PtdIns(3,4)*P*_*2*_, thereby inhibiting BCR responses [60].

Antigen challenge triggers a primary humoral immune response that is characterized by clonal expansion and B-cell differentiation into IgM-secreting plasma cells. Alternatively, B cells can be recruited into the T-cell-rich areas of the splenic follicles, where the Ig loci undergo class-switch recombination (CSR) and somatic hypermutation leading to the production of higher affinity antibodies of the IgG or IgE subclasses. Immunized p110δ-deficient mice show reduced germinal centre (GC) formation and impaired T-cell-dependent and T-cell-independent immune responses, suggesting a positive role for p110δ in the GC reactions [6,8]. However, there is also evidence for a negative role of p110δ in the GC reaction: Pten-deficient B cells fail to induce activation-induced cytidine deaminase, an essential regulator of CSR [61]. Moreover, Akt phosphorylation and the inactivation of Foxo proteins by p110δ was shown to suppress CSR, favouring the formation of IgM-secreting antibody effectors [62]. How these results correlate with the diminished IgG-mediated responses in p110δ-deficient mice is unclear. One possibility is that p110δ-deficient T cells cannot support full GC reactions. In addition, an earlier developmental lesion in p110δ-deficient B cells might reduce dramatically the number of B cells poised for participation in the GC reaction. Therefore, although p110δ has a positive role in mitogenic signalling through the BCR, p110δ might also negatively regulate differentiation programmes that lead to CSR and secondary immune responses.

## p110δ in T-cell development and function

T-cell development in the thymus progresses through three checkpoints: β-selection, where CD4^–^CD8^–^ double-negative T cells are examined for pre-TCRβ expression; positive selection, where CD4^+^CD8^+^double-positive TCRαβ^+^ T cells are selected to become CD4^+^ or CD8^+^ single-positive T cells; and negative selection, where autoreactive T cells are eliminated.

PtdIns(3,4,5)*P*_3_ signalling is both sufficient and necessary for β-selection. Lck–Cre-mediated conditional deletion of Pten in the T-cell lineage enables the development of double-positive thymocytes in the absence of pre-TCR expression [63,64]. Similarly, in the presence of artificially high expression of its ligand CD86, CD28 can promote the development of double-positive T cells in the absence of pre-TCR, but only if the PI3K-binding motif is intact [65].

β-selection seems normal in p110δ-deficient mice [8,66], suggesting the involvement of other class I PI3Ks. One of these might be p110γ, as T cells lacking this PI3K have a partial defect at this selection stage, resulting in a reduced number of double-positive T cells [4]. p110γ^−/−^ p110δ^−/−^ double-knockout mice have a profound block at the pre-TCR selection step, with a dramatic reduction of double-positive cells [66,67]. This is a surprising observation, given that GPCRs have not previously been shown to be required at this selection step. It is presently unclear whether there is a chemokine receptor or another GPCR that functions in concert with the pre-TCR at this stage, or whether p110γ can be activated by non-GPCR receptors. Positive selection is unaffected in p110δ-deficient thymocytes; however, negative selection of autoreactive T cells is partially impaired [68].

In peripheral T cells, PI3Ks can be activated by the TCR, by costimulatory receptors such as CD28 and ICOS, and by receptors for cytokines and chemokines [30,69]. p110δ seems to be the main PI3K isoform that generates PtdIns(3,4,5)*P*_3_ downstream of the TCR and CD28, although p110α and/or p110β are also likely to contribute [8,14,70]. In the immune synapse, PtdIns(3,4,5)*P*_3_ accumulation is observed as one of the earliest traceable signals and is sustained for hours, as long as the conjugate formation between antigen-presenting cells and T cells is maintained [33–35]. The relative contributions made by the TCR and CD28 to PI3K activity in the synapse are still unknown; however, synapse accumulation can occur under conditions where CD28 costimulation is absent [33,37]. A key effect of PI3K signalling in T cells is the activation of Akt, which phosphorylates Foxo transcription factors that then become excluded from the nucleus [14,38,71]. In addition, Akt can contribute to glycolysis and protein synthesis; however, PI3K-independent pathways can also contribute to at least some of these effects [38,72–74].

In T cells with inactive p110δ, defects in proliferation and cytokine secretion were most clearly revealed when TCR-transgenic T cells were stimulated with cognate antigen [8,14]. In particular, T-helper (Th)1 and Th2 cytokine production was reduced dramatically [14]. As a consequence of reduced Th2 responses, p110-deficient mice were protected from experimentally induced airway inflammation [75]. *In vitro*, the reduction in Th1 and Th2 cytokine production could not be rescued by providing an excess of exogenous cytokines and was still defective among T cells that had undergone several rounds of division [14]. Therefore, there seems to be a block in the genetic programme that enables T cells to open the IL-4 or interferon (IFN)γ gene loci. We hypothesize that this block reflects the capacity of PI3Ks to relieve T cells from the blocks imposed by transcription factors of the Foxo family. Unless the suppression by Foxo transcription factors is lifted by PI3K activation, the cell is unable to differentiate further. In this scenario, PI3Ks and, more specifically, p110δ, function in parallel to the canonical TCR signalling pathway initiated by PLCγ (Figure 1). That is, initial activation events that lead to clonal expansion and IL-2 secretion do occur but further differentiation is blocked. Consistent with this notion, mice that lack Foxo3a, one of the Foxo transcription factors, suffer from exaggerated Th1 and Th2 responses and autoimmune syndromes [76].

A third lineage of CD4^+^ T cells, referred to as regulatory T (Treg) cells, restrict the expansion and function of Th cells [77]. Mice that lack Treg cells die young as a consequence of T cell-mediated multi-organ destruction. Mice that have partial defects in Treg-cell development tend to develop colitis as Treg cells have a key role in suppressing immune responses against the gut flora [78]. p110δ-deficient mice also show subclinical signs of colitis as detected by histological examination [8]. Moreover, p110δ-deficient Treg cells show attenuated capacity to suppress Th cells *in vitro* and fail to protect against experimentally induced colitis *in vivo* [68]. Interestingly, p85β knockout mice with T-cell-specific deletion of p85α have reduced proportions of Treg cells, show signs of colitis and develop an autoimmune disease that is reminiscent of Sjögren's syndrome [79]. The latter experiments also demonstrated that although p85α-deficiency is sufficient to suppress PI3K signalling in B cells, both p85α and p85β need to be deleted to uncover a PI3K-deficient phenotype in T cells [93].

## p110δ in mast cell development and function

Mast cells are amongst a select group of cells (including basophils and eosinophils) that express the FcɛRI (Figure 2). Antigen-specific IgE from the plasma binds to FcɛRI with high affinity, enabling the mast cells to participate in the adaptive immune response. Antigen-induced aggregation of FcɛRI-bound IgE activates a series of intracellular signalling events, culminating in secretory-granule exocytosis and the release of pro-inflammatory mediators that promote the allergic cascade [19].

The activation of FcɛRI triggers a tyrosine kinase cascade involving Lyn, Fyn and Syk, resulting in the activation of the linker for activation of T cells (LAT)–PLCγ and GRB2-associated binding protein 2 (GAB2)–PI3K pathways (Figure 2) [19]. These pathways recruit PI3K and interact with effectors that drive Ca^2+^ mobilization and PKC activation, which are both a prerequisite for mast-cell exocytosis [80].

Pan-PI3K inhibitors can attenuate IgE–antigen-dependent secretory-granule exocytosis severely [81]. Conversely, genetic interference with SHIP and Pten enhances FcɛRI responses [82,83]. Akt might control IL-2 and TNF-α production by regulating NF-κB [84]. Two other potential PI3K effectors downstream of FcɛRI in mast cells are Btk and phospholipase D1 (PLD_1_), which both influence Ca^2+^ mobilization and PKC activation strongly. Btk enhances the activities of PLCγ and PKCβ1 (the latter directly, or indirectly through the regulation of PLCγ) [19]. Deletion or mutation of the Btk PH domain (as observed in Xid mice) results in reduced FcɛRI-dependent Ca^2+^ signalling, degranulation and cytokine release [19]. PLD_1_ contains a PtdIns(3,4,5)*P*_3_-interacting PX domain [85], and its activation leads to the production of phosphatidic acid, which is metabolized further into an important source of DAG that can activate PKC and might provide an additional link between the PI3K and the PLCγ pathway in augmenting the PKC activation [86].

Most PI3K-dependent activity downstream of the FcɛRI is mediated by p110δ, and antigen receptor-induced Akt activation is almost completely eliminated following p110δ inactivation [10]. Mast cells derived from p110δ-deficient mice, or wild-type mast cells treated with a p110δ-selective inhibitor, have a substantial reduction in IgE–antigen-dependent exocytosis and release of the pro-inflammatory cytokines TNF-α and IL-6 [10].

Another important pathway for regulating mast-cell proliferation, migration and adhesion is through the c-kit receptor Tyr kinase, which is activated by the SCF ligand. c-kit has a YxxM motif that can recruit p85–p110 complexes. Interestingly, similar to the FcɛRI, PI3K-dependent responses downstream of the c-kit receptor are almost completely p110δ-dependent [10]. Allergic responses occur in an SCF-rich environment, which potentiates FcɛRI responses. The observation that both the FcɛRI and its key modulating receptor c-kit depend heavily on p110δ activity is an important rationale for developing p110δ inhibitors for allergy indications [10]. Indeed, inactivating p110δ in mice results in a substantial reduction in the allergic response [10,11]. However, a residual PI3K-dependent allergic response is observed in p110δ-deficient mast cells and mice [10]. The identity of this PI3K(s) is still elusive and could either be another class 1A PI3K isoform or it could be p110γ. Indeed, GPCR-coupled p110γ is thought to operate downstream of activated FcɛRI through a GPCR-dependent autocrine or paracrine amplification mechanism [5].

## Concluding remarks

p110δ seems to have evolved as the main source of PtdIns(3,4,5)*P*_3_ production following antigen recognition by B cells, T cells and mast cells. Although the p110α and p110β isoforms might also contribute to antigen receptor signalling, their contributions seem less important than those of p110δ, for reasons that are not understood. In this respect, the role of p110δ is unique: its attenuation affects antigen receptor signalling in B cells, T cells and mast cells, yet p110δ is dispensable for the function of most tissues and organs. No other kinase associated with the antigen receptor signalling machinery shares this profile. Instead, the expression of Src, Syk and Tec family kinase isoforms differs between B cells, T cells and mast cells. Hence, p110δ presents a unique opportunity to modulate antigen receptor signalling using small-molecule inhibitors.

It will also be important to investigate the contribution of each of the four class I PI3K isoforms to signalling by other immune receptors. This should be facilitated through the availability of a range of PI3K gene-targeted mice and PI3K isoform-selective inhibitors that are now becoming available. These studies should pave the way towards the clinical development of small-molecule inhibitors against PI3K isoforms, in particular p110δ, which could alleviate harmful immune responses against self antigens, transplantation antigens and innocuous foreign antigens in allergy.

## Footnotes

† We have previously commented on the relative merits of different gene-targeting strategies [13] and in this review, we will refer collectively to p110δ^−/−^ and p110δ^D910A/D910A^ mice as p110δ-deficient.
